# Adaptations to implementation frameworks for minority ethnic groups to improve health equity: systematic scoping review

**DOI:** 10.1192/bjo.2025.10075

**Published:** 2025-08-08

**Authors:** Emma Mckenzie, Phoebe Barnett, Georgie Parker, Stephen Pilling

**Affiliations:** Centre for Outcomes Research and Effectiveness, Research Department of Clinical, Educational and Health Psychology, University College London, London, UK

**Keywords:** Health equity, ethnic inequities, implementation frameworks, cultural adaptations, scoping review

## Abstract

**Background:**

There are critical gaps within implementation science concerning health equity, particularly for minoritised ethnic groups. Implementation framework adaptations are important to facilitate health equity, which is especially relevant for psychiatry due to ethnic inequities in mental health; however, the range of potential adaptations has yet to be synthesised.

**Aims:**

This systematic scoping review aimed to identify and map the characteristics of adaptations to implementation frameworks for minority ethnic groups to improve health equity.

**Method:**

Bibliographic searches of the MEDLINE, Embase, PsycINFO and CINAHL databases were conducted, spanning the period from 2004 to February 2024 for descriptions of implementation frameworks adapted for minority ethnic groups. The characteristics of those meeting the criteria were narratively synthesised.

**Results:**

Of the 2947 papers screened, six met the eligibility criteria. Three different types of implementation frameworks were adapted across the six papers: evaluation, process and determinant frameworks. Most of the adaptations were made by expanding the original framework, and by integrating it with another model, theory or framework with an equity focus. The adaptations primarily focused on putting equity at the forefront of all stages of implementation from intervention selection to implementation sustainability. No studies measured the effectiveness of the adapted framework.

**Conclusions:**

The findings demonstrate that implementation frameworks are modifiable, and different elements can be adapted according to the implementation framework type. This review provides a starting point for how researchers and healthcare providers can adapt existing implementation frameworks to promote health equity for minoritised groups across a range of healthcare settings.

Implementation science was developed in direct response to the research to practice gap; the field aims to promote the translation and uptake of evidence-based interventions.^1^ It has long been recognised that getting research into practice is convoluted and difficult,^2^ implementation science is important for the uptake of research and other evidence-based practices into routine practice and ultimately improving the effectiveness and quality of health services.^3^ Despite its aims and the potential of implementation science, critical gaps remain within the field to facilitate health equity, and health disparities continue to be commonplace.^1,4^ Health disparities have been defined as significant differences between groups in healthcare access, quality or outcomes which are not due to selection bias.^4^ Most of the existing implementation science theories, models and frameworks (TMFs) do not address issues of power, structural racism and inequality that are pivotal to tackling these health disparities.^5^

During implementation, evidence-based interventions are often implemented generically across populations and care systems. As a result, the importance of unique contextual factors that reinforce health inequities are often overlooked. Furthermore, the historical, social and political forces that shape the delivery of interventions are typically ignored during implementation.^6–8^ This leads to the inequitable implementation of interventions across various settings and populations which skews application of the best available practices towards communities and organisations with high capacity and resources. When this occurs, it can further exacerbate health disparities based on race, ethnicity, gender, sexual orientation, socioeconomic status, etc.^9^

It is important to note that hospitals and healthcare settings are complex systems.^10^ Complex interventions introduced into complex systems present various challenges such as the active elements of the intervention being subject to more variation, as outcomes occur at the individual, population and/or system levels.^10,11^ The goal of implementation science is not to establish the health impact of an innovation or intervention, but to identify the factors that affect its uptake into routine practice.^12^ Implementation research investigates strategies to adopt and integrate evidence-based health interventions into clinical practice to improve patient outcome.^13^ However, getting evidence into practice for minority ethnic groups using implementation research may be challenging due to the under-representation of minority ethnic groups in health and social care research.^14^ This may explain the dearth in evidence regarding the unique implementation considerations for specific minority ethnic groups.

Implementation frameworks are important for translating evidence into practice, providing a structure for (a) describing and/or guiding the process of translating interventions and evidence into practice (process frameworks), (b) analysing what influences implementation outcomes (determinant frameworks) and (c) evaluating an implementation effort (outcome frameworks).^15^ Researchers have warned that suboptimal use of implementation frameworks can slow the translation of evidence into practice, cause stakeholders to misjudge their implementation context and develop inappropriate implementation strategies, and ultimately limit the public health impact of an implementation effort.^15^ However, the frameworks themselves seem inherently suboptimal because despite the large repertoire of existing implementation frameworks, the majority do not explicitly mention or address factors such as power, inequality, health equity or racism.^4,5^ Therefore, the public health impact of implementation efforts which employ these frameworks may be limited for particular groups, even when they are used optimally.

There is a dearth of implementation frameworks which consider the unique contextual factors and needs of different groups; this may contribute to the aforementioned inequities that are commonplace during implementation efforts. Current implementation frameworks are broad and do not consider the needs of minoritised groups,^16^ or the larger social, historical, economic and political issues. This causes long-standing concerns for cultural and ethnic minority groups, such as systemic racism, to be ignored.^17^ By not specifying or considering the needs of minoritised groups, implementation frameworks assume that the needs of all patients and clinicians are the same. This false assumption is not only a potential implementation barrier, but it can perpetuate hidden bias within large systems and contribute to the ongoing oppression of minority ethnic populations.^18^ This is especially important for those working in mental health settings to be aware of and to understand, because a wealth of literature already shows that people from minority ethnic groups are disproportionately affected by poor mental health, are at increased risk of specific mental illnesses and are over-represented in the most restrictive aspects of mental health care such as involuntary admissions and restrictive practices.^19–22^ Therefore, specifying and considering the needs of minoritised groups when implementing innovations/change in mental health care is particularly important to avoid exacerbating existing health inequities in psychiatric disorders and treatment.

Moving the field of implementation science to a place where health equity considerations are foundational is crucial to strengthening the field and enhancing its impact.^1^ There are growing calls to the field to prioritise racial and ethnic equity approaches and to explicitly tackle racial disparities in healthcare.^1,23^ Researchers have begun to take an interest in these issues.^23–25^ Theoretical and empirical adaptations to implementation frameworks have been made to ask explicit questions about racial equity in health and to improve health equity for minority ethnic groups during intervention implementation.^26^ A recent scoping review conducted by Gustafson et al^27^ identified and analysed equity-focused implementation science TMFs to see how they have been or may be used to address ethnic health inequalities. They found 15 TMFs; of these, only three were established TMFs which had been adapted in an equity context. This highlights that only a small portion of the existing equity-focused implementation TMFs are adaptations of established TMFs. As Baumann and Cabassa^6^ point out, a plethora of implementation frameworks already exist, therefore the creation of more is not necessary; they posit that what is required instead, is an understanding of how to adapt and apply existing frameworks to address ethnic inequities in healthcare. Napoles, Santoyo-Olsson and Stewart^16^ argue that publishing information on such adaptations, including methods and results, will advance implementation research and promote health equity in these communities.

To our knowledge, no previous scoping or systematic reviews have been conducted mapping the characteristics of adapted implementation frameworks for minority ethnic groups. Building on the work of Gustafson et al,^27^ this review aims to address an important evidence gap by describing the characteristics of adaptations made to existing implementation frameworks for minority ethnic groups. In doing so, we hope to further the understanding of how to utilise established implementation frameworks to meet the needs of specific populations, in turn addressing health inequalities.

The review sought to address the following questions:

- What are the characteristics of the adaptations that have been described and/or applied to implementation frameworks for minority ethnic groups to improve health equity during healthcare intervention implementation?
- How many of these studies have looked at the effectiveness of the adapted implementation framework and what measures of effectiveness have they used?

## Method

This scoping review followed a pre-specified protocol which was registered *a priori* (https://doi.org/10.17605/OSF.IO/EVYDF), and guidance on the conduct and reporting of scoping reviews.^28^ The Preferred Reporting Items for Systematic Reviews and Meta-Analyses extension for Scoping Reviews (PRISMA-ScR) checklist^29^ was used to guide the reporting of the results for this review (Supplementary material Additional file 1 available at https://doi.org/10.1192/bjo.2025.10075). The review adhered to the registered protocol, with the exception of the following: at the full text screening stage 100% of references were double screened instead of the 10% stated in the protocol.

### Eligibility criteria

We considered papers meeting the following criteria to be included in the review:

#### Participants

- Any age group
- Ethnic minorities, migrants, refugees, indigenous minorities and people referred to or defined as belonging to a racial or ethnic ‘minority group’
- People with a suspected or a diagnosed health problem, people experiencing symptoms of a health condition or people seeking or accessing healthcare. Both mental health and physical health conditions were included.

#### Concept

Papers needed to describe, apply and/or evaluate implementation frameworks which have been adapted to improve health equity for people from minority ethnic groups. We defined this as follows:

- Implementation was defined as the process of integrating or putting to use innovations and interventions within a health setting.^30,31^
- Framework was defined according to the definition provided by Moullin, Dickson^15^ which is narrative or graphical representations of the key factors, concepts or variables of implementation. All five categories of Nilsen’s^32^ taxonomy of implementation frameworks were included; process models, determinant frameworks, classic theories, implementation theories and evaluation frameworks. These are defined as the following: process models describe and/or guide the process of translating research into practice; determinant frameworks, classic theories and implementation theories all try to understand and/or explain what influences implementation outcomes; and finally, evaluation frameworks provide a structure for evaluating implementation endeavours.^32^
- Adaptation was defined as any described or applied modification to an existing implementation framework. This included any newly developed implementation frameworks based on an existing implementation framework or combination of frameworks. Papers which applied these adapted frameworks, those that empirically measured the effectiveness of these adapted frameworks and those that did neither of these things were all eligible.
- Health equity was defined as everyone having a fair and just opportunity to be as healthy as possible.^33^ Improvement in health equity was defined according to Braveman et al’s^33^ definition of how progress toward health equity is measured: reducing and eliminating disparities in health and in the determinants of health that adversely affect marginalised groups, specifically minority ethnic groups in this review. Health equity improvement in this review also included papers which highlight the health needs and inequities faced by ethnic minority groups as a first step toward reducing disparities.

#### Context

Papers related to any physical or mental health condition, innovation or intervention were included. Any health system and any healthcare settings including community and primary care in middle-high income countries were included in this review.

#### Types of sources

All study designs and publication types were included in this review. This included theoretical and opinion papers which described a modification to an implementation framework without empirically testing it. Only papers published in the English language were included due to the technicalities in implementation framework terminology. The inclusion was limited to papers published since 2004 as the field of implementation science was only developed in the early 2000s^34^ and the first dedicated peer-review journal for the field was established in 2006.

### Search strategy

We searched the MEDLINE, Embase, PsycINFO and CINAHL databases for published literature and the HMIC and Global Health databases for grey literature from 2004 to 16th February 2024. The search strategy included both keyword and subject heading searches and was supplemented by hand searching reference lists of relevant papers identified through the search process. The full search strategy is available in Supplementary material Additional file 2.

### Selection of sources of evidence

The records retrieved were downloaded and deduplicated using Endnote Version 20 for macOS (Clarivate, London, UK; https://support.clarivate.com/Endnote/s/article/Download-EndNote?language=en_US). The title and abstract of all identified records were then screened by one researcher (E.M.) according to the predetermined inclusion criteria. A second researcher (P.B.) dually and independently screened 10% of references to ensure that the inclusion criteria was applied uniformly. There was 90% agreement. Disagreements were resolved by reviewing the abstract together and discussing, and in any cases where discrepancies remained – where one reviewer considered the reference ineligible and one considered it eligible, the full text was reviewed. The inclusion criteria was then discussed further to ensure consensus. Following this, a further 15% of references were then independently double screened by PB and there was 100% agreement for these additional references, suggesting that this further discussion led to greater consensus. Overall, in total, 25% of references were dual screened at the title and abstract stage. The full text of all remaining sources of evidence were then assessed in detail against the inclusion criteria by two researchers independently (E.M., P.B., G.P. or an MSc student (M.T.)). The reasons for exclusion of papers at this stage were recorded. Any disagreements between researchers were resolved through discussion or with an additional reviewer. Screening at the title and abstract stage was undertaken using the Rayyan web application for macOS (2014 release; Qatar Computing Research Institute, Qatar, Doha; http://rayyan.qcri.org)^35^ and at full text stage using Microsoft Excel for macOS. The reasons for any exclusion of items at full text are available in Supplementary material Additional file 3.

### Data extraction

A data extraction form was developed by two researchers using Microsoft Excel. The following data was extracted from all included papers: (a) participant characteristics – health condition, ethnicity, age and gender; (b) concept – implementation framework being adapted, type of framework, details of the adaptation described in paper and intervention/innovation; (c) context – country and health setting; (d) methods – type of paper, application of framework and conclusions, and (e) whether the effectiveness of the framework was measured. Two researchers (E.M. and G.P.) independently extracted the data from the included papers into the data extraction form. There were some minor discrepancies in the extracted data which were highlighted, discussed and resolved.

### Critical appraisal

The review intended to use the Mixed Method Appraisal Tool (MMAT)^36^ to critically appraise evaluation studies which had empirically tested the effectiveness of the adapted implementation framework. However, as no evaluation studies were found, the Mixed Method Appraisal Tool was not used.

### Data analysis

The data from all included papers were extracted and presented in tables. The tables collated and summarised the characteristics of each paper and the different types of implementation frameworks represented across papers. To inform research question a, the different types of adaptations reported were described. Adaptations were described according to (a) the descriptions of the modifications stated in the paper, (b) the method of adapting the framework i.e. whether there was an integration with other frameworks/disciplines and (c) the authors’ conclusion of the adapted framework that they presented.

Research question 2 was answered in narrative form as we did not find evidence of studies that measured the effectiveness of the adapted implementation frameworks. A narrative summary of the evidence was presented to accompany the tabulated results and to describe how the data extracted from all included papers related to the review objectives and questions.

## Results

**Fig. 1:**
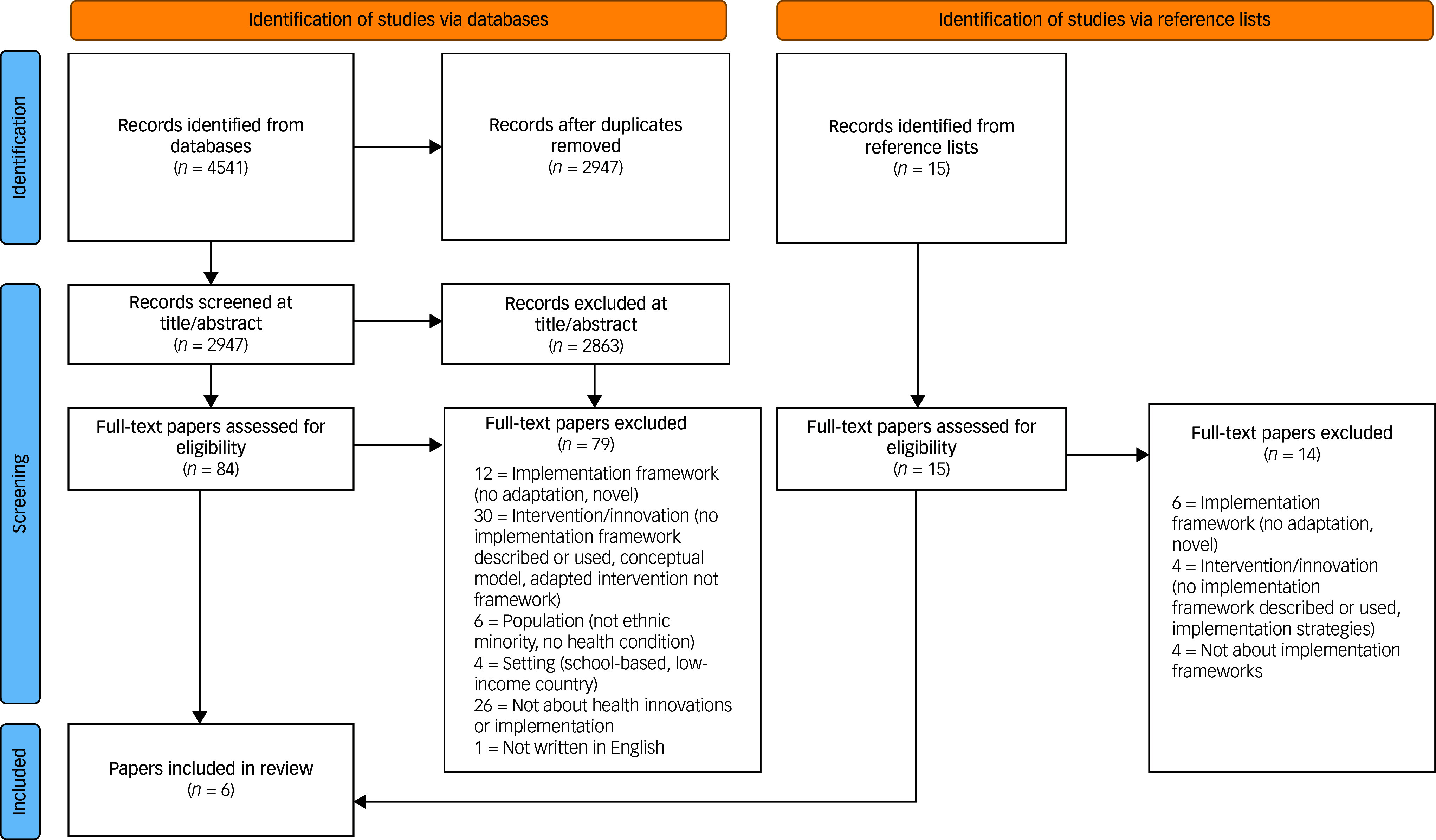
PRISMA flow diagram of paper selection.^40^

The search identified a total of 2947 non-duplicate papers and an additional 15 papers from reference list searches. Six out of 99 papers reviewed in full text met the inclusion criteria and were included in the review. The following implementation frameworks were adapted across these six papers: Proctor’s Framework,^6^ The Interactive Systems Framework,^16^ Integrated-Promoting Action on Research Implementation in Health Services,^4^ Consolidated Framework for Implementation Research (CFIR),^37^ The Equity-based Framework for Implementation Research^38^ and RE-AIM (reach, effectiveness, adoption, implementation and maintenance).^39^ A PRISMA flow diagram of the study selection is presented in Fig. 1.

### Characteristics of papers

#### Participant characteristics

All the included papers outlined the ethnic groups that the adapted implementation framework they described can be applied to. Across the papers this was outlined as being for ethnic minority populations; however two of the papers went further and adapted a framework with a focus on a specific ethnic minority group – in one paper this was Black people in America^4^ and in the other paper this was the Māori Indigenous people of New Zealand.^38^ Two out of six of the papers also adapted the framework with a specific age and gender. Four out of six papers were non-specific about age and gender and only specified ethnicity. All four of these papers^6,16,38,39^ were theoretical papers which described the adapted framework without applying it. The paper by Senier et al^37^ was also a theoretical paper which did not apply the framework; however, the framework was described with a specific demographic group that it could be applied to. Four out of six of the papers^6,16,38,39^ did not specify a particular health condition that the adapted framework could be used for. In the remaining two^4,37^ the conditions specified were physical health conditions.

#### Context

Three of the papers specified the country that the adapted implementation framework could be used in; in two of these this was the USA and in one paper this was New Zealand. In the remaining papers, the target country was not specified, although the authors were based in high-income countries. A range of healthcare settings were covered.

#### Concept

Across the included papers, three types of implementation frameworks were adapted: determinant, process and evaluation frameworks. The full characteristics of each included paper are provided in Table 1.

**Table 1:** Characteristics of papers

	Participant characteristics					Context		Concept
Paper ID	Ethnicity	Age	Gender	Health condition	Country	Health setting	Intervention	Type of implementation framework
Baumann 2020^6^	Vulnerable populations including ethnic minorities	Not specified	Not specified	Not specified	Not specified	Healthcare delivery generally	Not specified	Evaluation framework
Shelton 2020^39^	Diverse populations	Not specified	Not specified	Not specified	Not specified	Population health	Not specified	Evaluation framework
Napoles 2013^16^	Vulnerable groups including racial/ethnic minorities	Not specified	Not specified	Not specified	Not specified	Behavioural interventions in community settings	Not specified	Process framework
Gustafson 2024^38^	Māori	Not specified	Not specified	Not specified	New Zealand	Mainstream health services	Not specified	Process framework
Woodward 2019^4^	Black	Older adults	Male	Hepatitis C virus (HCV) treatment	USA	US Department of Veteran Affairs healthcare services	Qualitative interviews with males diagnosed with HCV to examine treatment barriers and facilitators	Determinant framework
Senier 2019^37^	Racial and ethnic minorities	Adults	Female	Hereditary breast and ovarian cancer screening	USA	Health screening clinics	Genomic screening for high risk of adult-onset hereditary breast and ovarian cancers	Determinant framework

### Characteristics of frameworks

This review aimed to characterise the adaptations that have been made to implementation frameworks for minority ethnic groups. Across the included papers, the adaptations made to the process frameworks and the evaluation frameworks outline several phases/stages or steps to follow/consider during implementation or when evaluating an implementation effort.

Fewer adaptations were made to the determinant frameworks. For the two determinant frameworks that were adapted, the adaptations were not presented in numerical form or steps/phases. Instead, they extended the framework by adding specific determinants which may affect equity during implementation. In the i-PARIHS framework adaptation,^4^ this was by adding the clinical encounter to the ‘innovation level’ and adding societal influence to the ‘context level’. In the CFIR framework adaptation,^37^ this took the form of adding fundamental causes that affect healthcare delivery to the model’s ‘outer setting’. These additional determinants encourage, during implementation, the consideration of context-specific factors that can impact health disparities.

See Table 2 for a full description of the adaptations made to each framework.

**Table 2:** Description and characteristics of adaptations

Description of adaptation			Characteristics of adaptation
Framework type	Framework	Adaptation	
Evaluation framework	Proctor’s Framework	Reframed five of the implementation indicators within the framework by applying an equity lens: Focus on reach from the very beginning Design and select interventions for vulnerable populations with implementation in mind Implement what works and develop implementation strategies that can help reduce inequities in care Develop the science of adaptation Use an equity lens for implementation outcomes	**Description of adaptation:** Reframed the framework **Method:** Applied an equity lens/approach **Conclusion:** Put equity at the forefront of implementation Can be used in individual settings but it is also a call to the implementation science field generally
Evaluation framework	RE-AIM	An expansion of RE-AIM to enhance sustainability and increase health impact and health equity over time. Four main adaptations: Extension of ‘maintenance’ to include longer-term intervention sustainability and evolvability across the life of EBIs including adaptation and potential de-implementation in light of changing context, population needs and evidence Iterative application of RE-AIM assessments Explicit consideration of equity and costs as fundamental forces that need to be addressed across RE-AIM dimensions to enhance sustainability Use RE-AIM with other existing frameworks that address contextual factors and multi-level determinants of sustainability	**Description of adaptation:** Expanded the framework **Method:** Applied a health equity approach **Conclusion:** Reinforces the importance of promoting health equity within the context of implementation sustainability
Process framework	The Interactive Systems Framework (ISF)	Integrated and expanded both ISF and a model for adapting interventions to a new culture by Wainberg et al (2007)^41^ to develop seven iterative, nonlinear implementation phases for health disparity communities: Establish infrastructure for translation partnership Identify multiple inputs (information gathering) Review and distil information (synthesis) Adapt and integrate candidate programme components (translation) Build general and specific capacity (support system) Implement intervention (delivery system) Develop appropriate design and measures (evaluation)	**Description of adaptation:** Expanded the framework **Method:** Integrated the framework with a model for adapting evidence-based behavioural interventions to a new culture **Conclusion:** Integrates evidence-based interventions with community best practice whilst building local capacity to address disparities
Process framework	The Equity-based framework for Implementation Research	Worked in collaboration with Māori and consumer advisory groups and local health equity researchers and stakeholders to adapt the framework and make its foundations grounded in Te Tiriti o Waitangi and Te Ao Māori concepts, and to change the language to make it less research-focused. Five aspects of the framework were adapted: Changed the research-focused language to language that would allow the framework to be utilised across academic and service settings Inclusion of, or prompt for, community engagement and leadership, particularly in the implementation planning phase The addition of a clear ‘action’ step where implementation occurs, in order to encourage active equity-focused monitoring and feedback while the intervention is being implemented Incorporated an evaluation of intervention effectiveness Removal of the term ‘universal health coverage’ from the context, as it was not relevant within the local health system	**Description of adaptation:** Expanded the framework **Method:** Worked in collaboration with stakeholders to incorporate relevant principles valued by the ethnic minority group, and to make refinements that ensure the framework is useable across research and service settings **Conclusion:** The framework was adapted to the local context and grounded in the principles of the community
Determinant framework	Integrated-Promoting Action on Research Implementation in Health Services (i-PARIHS)	Integrated the i-PARIHS framework with the health care disparities framework which explains factors relevant to implementation and disparities in healthcare: Modified and extended the ‘innovation level’ of i-PARIHS to also include the clinical encounter which is described as the interaction between recipients and the innovation itself Extended the ‘context level’ in i-PARIHS by adding societal influence which is stated as especially important to consider when assessing all other factors because of the impact that society can have on healthcare disparities	**Description of adaptation:** Integrated the framework with a health disparities framework **Method:** Modifies the framework to better assess health equity determinants **Conclusion:** Developed a new framework – The Health Equity Implementation Framework
Determinant framework	Consolidated Framework for Implementation Research (CFIR)	Expands the area the CFIR call the ‘outer setting’ by adding fundamental causes that affect the way healthcare delivery unfolds. These fundamental causes are political, social and economic forces and include: Poverty and unemployment Limited access to services Neighbourhood segregation Geographical maldistribution of health services Inadequate health policies Limited healthcare funding Inequitable distribution of educational opportunities	**Description of adaptation:** Expanded the framework **Method:** Integrated the framework with a sociological theory of health inequities – The Fundamental Cause Theory **Conclusion:** Puts the anticipation of the potential for inequities at the centre of health implementation.

EBI, evidence-based interventions, programmes, practices and policies; RE-AIM, reach, effectiveness, adoption, implementation and maintenance.

### Characteristics of adaptations

The adaptation made to the implementation framework in four of the papers was characterised as an *expansion* to the framework, in one of the papers it was characterised as a *reframing* of the framework and in the final paper a *new framework* was developed.

In terms of the method of adapting the frameworks, the two papers which adapted evaluation frameworks *applied an equity approach* to the framework.^6,39^ One of the papers which adapted a process framework collaborated with the specific ethnic minority community of interest and with local health equity researchers to expand the framework by *incorporating* principles valued by the community and changing the language to allow the framework to be utilised across both academic and service settings.^38^

The other three papers *integrated* the framework with another model, theory or framework (MTF).^4,16,37^ The integrations in these three papers were with (a) a model for adapting evidence-based behavioural interventions to a new culture^16^ (b) a healthcare disparities framework which explains factors relevant to implementation and disparities in healthcare^4^ and (c) a sociological theory of health inequities – the fundamental cause theory.^37^ Adaptations through integration were conducted for both process and determinant frameworks, illustrating that this form of adaptation is suitable for different types of implementation frameworks. For all adaptations applied, the method of adapting the frameworks had an equity focus.

Across the included papers, the conclusions drawn following implementation framework adaptations can be characterised into the following three themes for each type of implementation framework. First, for evaluation frameworks, that it is important to keep equity at the forefront of all stages of implementation, from the initial design and selection of interventions to implementation sustainability.^6,39^ Second, for determinant frameworks, the main conclusion was that during implementation, potential inequities should be understood and anticipated, and the equity determinants assessed.^4,37^ Finally, for process frameworks, when implementing interventions in health-disparity communities, work should be conducted in partnership with the community to understand community principles and best practice, and support them by building their capacity to address disparities.^16,38^

### Measures of effectiveness

Although this review aimed to describe reports of the effectiveness of the adapted frameworks and what measures of effectiveness they had used, we did not find studies which looked at or measured the effectiveness of the adapted implementation framework that they presented. One paper,^4^ however, did conduct a preliminary study to assess how feasible it was for researchers to use the adapted framework, which involved applying the framework to design a qualitative interview guide and interpreting the results. They found that the framework was feasible and allowed barriers and facilitators to be identified at all levels; some barriers were generic implementation issues and others were unique to racial minority patients. This paper did not empirically measure the effectiveness of the framework.

## Discussion

This systematic scoping review identified six adapted implementation frameworks for minority ethnic groups to improve health equity. Regarding the key characteristics of the adaptations, most papers made adaptations by expanding the original framework, and by integrating it with another MTF with an equity focus. Across the six papers, three different types of implementation frameworks were adapted: evaluation, process and determinant.

Concerning the characteristics of the adaptations that were made to different types of implementation frameworks: evaluation frameworks added 4–5 phases which promote equity considerations at every stage of implementation – from intervention selection to evaluations of implementation sustainability. Process framework adaptations expanded the framework by either incorporating the relevant principles of the community or integrated it with a model for adapting interventions to new cultures. The adaptations that were made to the determinant frameworks were the addition of context-specific factors which may impact health disparities and need to be considered during health intervention implementations. Although there is considerable overlap between some of the implementation framework categories used in this review, and in the literature, these categories are not always recognised as separate types. It is important to recognise that implementation frameworks differ in their assumptions, aims and other characteristics, which have implications for their use,^32^ and similarly their adaptation method. The findings in this review demonstrate methods that future research can use to adapt different types of implementation frameworks.

The conclusions of the included papers call attention to the factors that different types of implementation framework could include to move towards more equitable health implementation. These were summarised as: for implementation processes to include working in partnership with communities to understand and incorporate community principles and best practice, and to build their capacity to address disparities; for evaluations implementations to measure equity across all stages of the intervention implementation; and, finally, for implementation determinants to include an understanding, assessment and anticipation of the potential equity and inequity determinants. The implementation science field has been predominantly equity-agnostic, taking the assumption that improvements in the quality and fidelity of implementation will also redress inequities.^42^ However, a dialogue is beginning about how to infuse an equity approach in implementation to proactively address health inequities among underserved communities.^6,23^ The conclusions of the included papers add to this dialogue and demonstrate potential ways to approach this. A recent study by Baumann et al^43^ assessing researchers capabilities, opportunities and motivation to conduct equity-oriented dissemination and implementation found that researchers have high levels of motivation to engage in equity-oriented implementation but many felt they lacked knowledge of equity-focused frameworks. The findings in this review collate the existing equity-oriented implementation frameworks and their learnings, which researchers can use.

When describing the target group for the adapted implementation framework most of the papers referred to ethnic minorities generally. Only two papers specified a minority ethnic group and outlined how the adapted framework could improve health equity for this group. Implementation research should be cautious about grouping minority ethnic groups together during studies of population needs, as ethnic minorities are not a homogenous group and research shows they experience different health inequalities;^19^ therefore, taking a generic ‘minority ethnic group’ approach may reinforce the same inequities that the research aims to address. However, it is worth noting that in the included papers, the adaptations to the frameworks are framed flexibly, in a way that allows them to be tailored to any specified minority ethnic group, if any additional context and information is considered.

Within the literature there are examples of frameworks created for use in specific ethnic communities. This includes the He Pikinga Waiora (HPW) Implementation Framework^44^ which was developed in particular for Māori communities and is grounded in Indigenous critical theory. Each element is consistent with Kaupapa Māori aspirations. Subsequent applications and studies of this framework have found that the HPW implementation framework is a comprehensive model for understanding implementation effectiveness for Māori and Indigenous communities in general.^45–47^ The HPW was not included in this review because it is a novel framework and therefore did not meet the inclusion criteria of an adaptation of a pre-existing implementation framework. Nevertheless, it represents adjacent methods that implementation researchers are using to improve health equity for minority ethnic groups – working in partnership to create novel frameworks grounded in the culture of the community. Another method is the combination of existing implementation frameworks with participatory approaches. Puthoopparambil, Phelan^48^ combined the Normalisation Process Theory implementation framework with Participatory Learning and Action research and gained new insights into macro-level influences on the implementation of trained interpreters in health settings. These adjacent methods are important to consider. Adapted implementation frameworks are beneficial because they build on what is already available and known to be effective, but the needs of some groups may mean that there is little relevance in the original frameworks and in these instances the development of a framework from scratch may be more beneficial for improving equity.

The findings in this scoping review of the literature highlight the emerging adaptations to implementation frameworks for minority ethnic groups. The literature shows that this can go even further – adapted implementation frameworks themselves, can be adapted for more specific populations. For example, one of the adapted implementation frameworks included in this review, the Health Equity Implementation Framework,^4^ was adapted further by Gustavson et al^49^ to advance the equitable implementation of aging innovations for older-adult minority ethnic populations – suggesting that there is no end point to adaptations of implementation frameworks. Adaptations to implementation frameworks for minority ethnic groups have also been conducted in school-based implementations to promote equity,^26,50^ demonstrating that this method of promoting equity is not limited to healthcare interventions either.

The healthcare interventions examined in the papers in this scoping review were focused on physical health; none of the papers looked specifically at mental health interventions or settings. This finding was surprising given the wealth of literature showing the long-standing health inequities in psychiatry that people from minority ethnic groups experience.^19,20^ In the UK, compared to the majority group, people from minority ethnic groups are more likely to have undiagnosed mental illness, come into services via crisis and receive a diagnosis of severe mental illness. Research shows that these contrasting patterns of service access and utilisation incur significant personal costs but also healthcare costs.^51^ This illustrates how consequential the study and identification of adapted implementation frameworks for minority ethnic groups in mental health interventions and settings could be – from both a health equity perspective and an economic perspective. There has been a notable absence of progress in addressing mental healthcare inequalities for minority ethnic communities.^51–53^ If it is found that adapting the implementation of interventions to the needs of minority ethnic groups has economic benefits, mental health services in the UK – which operate under financial constraints,^54^ may be incentivised to make progress in addressing ethnic inequalities by adapting intervention implementation as standard practice. Future studies into adapted implementation frameworks in mental health settings should therefore study the economic consequences in addition to the health equity consequences.

None of the papers included in this review measured the effectiveness of the adapted implementation framework that they developed. This finding was unsurprising as research shows that instruments to measure implementation outcomes are underdeveloped, with few available instruments and the limited psychometric quality of existing instruments.^55^ Furthermore, the majority of existing implementation outcome instruments have been developed to measure the implementation of specific interventions.^56^ There seems to be a dearth of instruments and routine practice of measuring and validating the actual implementation frameworks themselves. Indeed, a review of implementation frameworks for the telehealth service by Van Dyk^57^ found that the validation of implementation frameworks is scant and concluded that future research should consider the development of implementation framework effectiveness and validation approaches.

### Strengths and limitations of review

This review has several strengths. This is the first paper, to our knowledge, to map the characteristics of the adaptations that are being made to implementation frameworks for minority ethnic groups to improve health equity. In doing so, this paper directly responds to recent calls to the field to find ways to infuse an equity approach in implementation and ultimately adds to the advancement of the Implementation Science field. A plethora of implementation frameworks already exist; this review demonstrates ways to modify these frameworks to make them more equitable for minority groups rather than adding to the large number of frameworks in existence. Another strength of this review is that the analysis specified the adaptations and recommended considerations for frameworks according to their implementation framework type. This is an accessible way for future practitioners to see how they can adapt their implementation efforts, and future research into implementation framework adaptations can add to this typology of adaptations and analyse them further.

This review has several limitations. While the full texts of all eligible studies were independently double screened, we did not double screen all papers at the title and abstract screening stage. Another one of the limitations of this review is that not all types of implementation frameworks were represented in the included papers, for example classic theories, therefore this paper is not able to illustrate the adaptations that can be made to *all* types of implementation frameworks. In addition, the small number of included papers limits the scope to which the findings can be generalised to other implementation frameworks. Our inclusion criteria of adaptations made specifically for ethnic minority groups excluded papers such as Stanton et al’s^58^ in which implementation framework adaptations were made to promote equity among a range of minority groups. Furthermore, as none of the papers included in this review measured the effectiveness of the adaptations, it is not possible to ascertain how effective the adaptations found are at reducing health inequities for minority ethnic groups. In addition, the majority of the papers identified did not include an intervention for implementation when outlining the implementation framework adaptation; therefore, this limits the ability to investigate whether the evidence base and challenges for interventions vary for specific minority groups. Future research is needed to expand the findings in this initial scoping review and look more broadly at evidenced interventions and how to implement them, e.g. cultural competence training or co-design and participatory processes. Finally, the findings in this review are limited by the fact that only middle-high income countries were included. The adaptations found may not apply in low-income countries where healthcare systems are organised and financed differently. In addition, the factors that affect healthcare implementation in middle-high income countries are likely to be very different to low-income countries.

### Future directions

Implementation science must be proactive and consider measures to address health inequity among minority and historically underserved populations. This review demonstrates that three different types of implementation frameworks can be adapted to better suit the needs of ethnic minority groups in a healthcare setting. Routine adaptation of frameworks could be an important measure to combat the frequent broad untailored implementation of health interventions which has been commonplace in the field to date, and which overlook the importance of unique contextual factors and perpetuate health disparities. Current adaptations suggest that understanding and anticipating potential inequities for minority ethnic groups and keeping equity at the forefront during all stages of implementation could address this. Such adaptations can also be made for groups who are minoritised or marginalised in other ways, beyond race. Future research should empirically measure the effectiveness of the adapted implementation frameworks presented and advance the findings in this review by developing a typology of empirically tested adaptations to implementation frameworks for various minority groups.

## Supporting information

Mckenzie et al. supplementary material 1 Mckenzie et al. supplementary material

Mckenzie et al. supplementary material 2 Mckenzie et al. supplementary material

Mckenzie et al. supplementary material 3 Mckenzie et al. supplementary material

## Data Availability

Data availability is not applicable to this paper as no new data were created or analysed in this study.

## Supplementary material

The supplementary material is available online at https://doi.org/10.1192/bjo.2025.10075
