# Supplementary material for: Adaptations to implementation frameworks for minority ethnic groups to improve health equity: systematic scoping review
Source: BJPsych Open. 2025 Aug 8;11(5):e173. doi: 10.1192/bjo.2025.10075 (PMC12451719; doi:10.1192/bjo.2025.10075)
Supplement: Mckenzie et al. supplementary material 2 — Mckenzie et al. supplementary material [file S2056472425100756sup002.docx]

Additional file 2

Scoping review searches

Embase 16/02/24

*[implementation framework]*

1. implementation science/
2. (implement* or conceptual) adj2 (framework* or model* or theor*).ti,ab,kf,kw.
3. (implement* or adopt*) adj2 (facilitat* or barrier* or challenge* or determin*).ti,ab,kf,kw.
4. 1 or 2 or 3

*[minority ethnic groups]*

1. Exp ethnic group/
2. minority group/ or Hispanic/
3. black person/
4. refugee/
5. indigenous people/
6. cultural factor/
7. multiculturalism/
8. emigrant/ or migrant/ or immigrant/
9. (BME or BAME or Asian* Or African or Caribbean* or afro-caribbean* or Bangladeshi* or Chinese or Indian* or Pakistani or somali* or Latin* or hispanic*).ti,ab,kf,kw.
10. (Black).ti.
11. (black adj3 patient? Or men or women or adult? Or famil* or child* or adolesce* or teen* or young* or youth? Or old* or elder* or people or person?).ti,ab,kf,kw.
12. (Minority adj2 (group? Or ethnic*)).ti,ab,kf,kw.
13. (emigrant* or immigrant* or migrant*).ti,ab,kf,kw
14. (refugee*).ti,ab,kf,kw.
15. 5 or 6 or 7 or 8 or 9 or 10 or 11 or 12 or 13 or 14 or 15 or 16 or 17 or 18

*[health equity]*

1. health equity/
2. health disparity/
3. (health*) adj2 (equal* or equit*).ti,ab,kf,kw.
4. (health*) adj2 (inequit* or inequalit* or disparit*).ti,ab,kf,kw.
5. 20 or 21 or 22 or 23
6. 4 and 19 and 24
7. limit 25 to (yr=”2004-Current”)

**Results 16/02/24: 1265**

MEDLINE 16/02/24

*[implementation framework]*

1. implementation science/
2. (implement* or conceptual) adj2 (framework* or model* or theor*).ti,ab,kf,kw.
3. (implement* or adopt*) adj2 (facilitat* or barrier* or challenge* or determin*).ti,ab,kf,kw.
4. 1 or 2 or 3

*[minority ethnic groups]*

1. Exp ethnicity/
2. Exp minority groups/ or “Hispanic or Latino”/
3. black people/
4. refugees/
5. indigenous peoples/
6. cross-cultural comparison/
7. cultural diversity/
8. emigrants/ or immigrants/
9. (BME or BAME or Asian* Or African or Caribbean* or afro-caribbean* or Bangladeshi* or Chinese or Indian* or Pakistani or somali* or Latin* or hispanic*).ti,ab,kf,kw.
10. (Black).ti.
11. (black adj3 patient? Or men or women or adult? Or famil* or child* or adolesce* or teen* or young* or youth? Or old* or elder* or people or person?).ti,ab,kf,kw.
12. (Minority adj2 (group? Or ethnic*)).ti,ab,kf,kw.
13. (emigrant* or immigrant* or migrant*).ti,ab,kf,kw
14. (refugee*).ti,ab,kf,kw.
15. 5 or 6 or 7 or 8 or 9 or 10 or 11 or 12 or 13 or 14 or 15 or 16 or 17 or 18

*[health equity]*

1. health equity/
2. healthcare disparities/
3. ((health*) adj2 (equal* or equit*)).ti,ab,kf,kw.
4. ((health*) adj2 (inequit* or inequalit* or disparit*)).ti,ab,kf,kw.
5. 20 or 21 or 22 or 23
6. 4 and 19 and 24
7. limit 25 to (yr=”2004-Current”)

**Results 16/02/24: 1058**

PsycINFO 16/02/24

*[implementation framework]*

1. intervention/
2. (implement* or conceptual) adj2 (framework* or model* or theor*).ti,ab,id.
3. (implement* or adopt*) adj2 (facilitat* or barrier* or challenge* or determin*).ti,ab,id.
4. 1 or 2 or 3

*[minority ethnic groups]*

1. “racial and ethnic groups”/
2. minority groups/ or “Latinos/Latinas”/
3. blacks/
4. refugees/
5. indigenous populations/
6. cross cultural differences/
7. cultural diversity/
8. immigration/ or human migration/
9. (BME or BAME or Asian* Or African or Caribbean* or afro-caribbean* or Bangladeshi* or Chinese or Indian* or Pakistani or somali* or Latin* or hispanic*).ti,ab,id.
10. (Black).ti.
11. (black adj3 patient? Or men or women or adult? Or famil* or child* or adolesce* or teen* or young* or youth? Or old* or elder* or people or person?).ti,ab,id.
12. (Minority adj2 (group? Or ethnic*)).ti,ab,id.
13. (emigrant* or immigrant* or migrant*).ti,ab,id
14. (refugee*).ti,ab,id.
15. 5 or 6 or 7 or 8 or 9 or 10 or 11 or 12 or 13 or 14 or 15 or 16 or 17 or 18

*[health equity]*

1. health disparities/
2. ((health*) adj2 (equal* or equit*)).ti,ab,id.
3. ((health*) adj2 (inequit* or inequalit* or disparit*)).ti,ab,id.
4. 20 or 21 or 22
5. 4 and 19 and 23
6. limit 24 to (yr=”2004-Current”)

**Results 16/02/24: 1501**

CINAHL 16/02/24

| **Search ID#** | **Query** | **Limiters/Expanders** |
| --- | --- | --- |
| S1 | MH “Implementation Science” | Search modes - Boolean/Phrase |
| S2 | TI ((implement* or conceptual) N2 (framework* or model* or theor*)) OR AB ((implement* or conceptual) N2 (framework* or model* or theor*)) | Search modes - Boolean/Phrase |
| S3 | TI ((implement* or adopt*) N2 (facilitat* or barrier* or challenge* or determin*)) OR AB ((implement* or adopt*) N2 (facilitat* or barrier* or challenge* or determin*)) | Search modes - Boolean/Phrase |
| S4 | S1 OR S2 OR S3 | Search modes - Boolean/Phrase |
| S5 | (MH “Ethnic Groups”) OR (MH “Black Persons”) OR (MH “Refugees”) OR (MH “Indigenous Peoples”) OR (MH “Cultural Diversity”) OR (MH “Immigrants+”) OR (MH “Hispanic Americans”) | Search modes - Boolean/Phrase |
| S6 | TI (BME or BAME or Asian* or African or Caribbean* or Afro-caribbean* or Bangladeshi* or Chinese or Indian* or Pakistani (or somali* or Latin* or hispanic*)) OR AB (BME or BAME or Asian* or African or Caribbean* or Afro-caribbean* or Bangladeshi* or Chinese or Indian* or Pakistani or somali* or Latin* or hispanic*) | Search modes - Boolean/Phrase |
| S7 | TI (black) | Search modes - Boolean/Phrase |
| S8 | TI ((black) N3 (patient? Or men or women or adult? Or famil* or child* or adolesce* or teen or young or youth? Or old* or elder* or people or person?)) OR AB ((black) N2 (patient? Or men or women or adult? Or adolesce* or teen or young or youth? Or old* or elder* or people or person?)) | Search modes - Boolean/Phrase |
| S9 | TI ((minority) N2 (group? Or ethnic*)) OR AB ((minority) N2 (group? Or ethnic*)) | Search modes - Boolean/Phrase |
| S10 | TI (emigrant* or immigrant* or migrant*) OR AB (emigrant* or immigrant* or migrant) | Search modes - Boolean/Phrase |
| S11 | TI (refugee*) OR AB (refugee*) | Search modes - Boolean/Phrase |
| S12 | S5 OR S6 OR S7 OR S8 OR S9 OR S10 OR S11 | Search modes - Boolean/Phrase |
| S13 | MH “Healthcare disparities” | Search modes - Boolean/Phrase |
| S14 | TI ((health*) N2 (equal* or equit*)) OR AB ((health*) N2 (equal* or equit*)) | Search modes - Boolean/Phrase |
| S15 | TI ((health*) N2 (inequit* or inequalit* or disparit*)) OR AB ((health*) N2 (inequit* or inequalit* or disparit*)) | Search modes - Boolean/Phrase |
| S16 | S13 OR S14 OR S15 | Search modes - Boolean/Phrase |
| S17 | S4 AND S12 AND S16 | Limiters = Date: 20040101-Current  Search modes - Boolean/Phrase |

**Results 16/02/24: 289**

Global Health 16/02/24

*[implementation framework]*

1. Implementation of research/
2. (implement* or conceptual) adj2 (framework* or model* or theor*).ti,ab.
3. (implement* or adopt*) adj2 (facilitat* or barrier* or challenge* or determin*).ti,ab.
4. 1 or 2 or 3

*[minority ethnic groups]*

1. Exp ethnic groups/
2. minorities/ or Hispanics/
3. black people/
4. refugees/
5. indigenous people/
6. cross cultural studies/
7. emigration/ or migrants/ or immigrants/
8. (BME or BAME or Asian* Or African or Caribbean* or afro-caribbean* or Bangladeshi* or Chinese or Indian* or Pakistani or somali* or Latin* or hispanic*).ti,ab.
9. (Black).ti.
10. (black adj3 patient? Or men or women or adult? Or famil* or child* or adolesce* or teen* or young* or youth? Or old* or elder* or people or person?).ti,ab.
11. (Minority adj2 (group? Or ethnic*)).ti,ab.
12. (emigrant* or immigrant* or migrant*).ti,ab.
13. (refugee*).ti,ab.
14. 5 or 6 or 7 or 8 or 9 or 10 or 11 or 12 or 13 or 14 or 15 or 16 or 17

*[health equity]*

1. health inequalities/
2. ((health*) adj2 (equal* or equit*)).ti,ab.
3. ((health*) adj2 (inequit* or inequalit* or disparit*)).ti,ab.
4. 19 or 20 or 21
5. 4 and 18 and 22
6. limit 23 to (yr=”2004-Current”)

**Results 16/02/24: 379**

HMIC 16/02/24

*[implementation framework]*

1. implementation/
2. (implement* or conceptual) adj2 (framework* or model* or theor*).ti,ab.
3. (implement* or adopt*) adj2 (facilitat* or barrier* or challenge* or determin*).ti,ab.
4. 1 or 2 or 3

*[minority ethnic groups]*

1. Exp ethnic minorities/
2. minority groups/
3. black people/
4. refugees/
5. cultural differences/
6. cultural pluralism/
7. emigration/ or migrants/ or immigrants/
8. (BME or BAME or Asian* Or African or Caribbean* or afro-caribbean* or Bangladeshi* or Chinese or Indian* or Pakistani or somali* or Latin* or hispanic*).ti,ab.
9. (Black).ti.
10. (black adj3 patient? Or men or women or adult? Or famil* or child* or adolesce* or teen* or young* or youth? Or old* or elder* or people or person?).ti,ab.
11. (Minority adj2 (group? Or ethnic*)).ti,ab.
12. (emigrant* or immigrant* or migrant*).ti,ab.
13. (refugee*).ti,ab.
14. 5 or 6 or 7 or 8 or 9 or 10 or 11 or 12 or 13 or 14 or 15 or 16 or 17

*[health equity]*

1. health inequalities/
2. equal opportunities/
3. ((health*) adj2 (equal* or equit*)).ti,ab.
4. ((health*) adj2 (inequit* or inequalit* or disparit*)).ti,ab.
5. 19 or 20 or 21 or 22
6. 4 and 18 and 23
7. limit 24 to (yr=”2004-Current”)

**Results 16/02/24: 49**
