# Supplementary material for: Adaptations to implementation frameworks for minority ethnic groups to improve health equity: systematic scoping review
Source: BJPsych Open. 2025 Aug 8;11(5):e173. doi: 10.1192/bjo.2025.10075 (PMC12451719; doi:10.1192/bjo.2025.10075)
Supplement: Mckenzie et al. supplementary material 3 — Mckenzie et al. supplementary material [file S2056472425100756sup003.docx]

Additional file 3

Reasons for exclusion of papers at full text screening

N=79

| **Reason for exclusion:** | **Number of papers excluded:** |
| --- | --- |
| **Intervention/innovation:** No implementation framework described or used, conceptual model or adapted intervention not framework | 30 |
| **Not a health innovation or implementation** | 26 |
| **Implementation framework:** No adaptation or novel framework | 12 |
| **Population:** Not ethnic minority or no health condition | 6 |
| **Setting:** School based or low-income country | 4 |
| **Paper not written in English** | 1 |
